# The regulation of lipid A biosynthesis

**DOI:** 10.1016/j.jbc.2025.110556

**Published:** 2025-07-31

**Authors:** Katherine R. Hummels

**Affiliations:** Department of Microbiology, University of Georgia, Athens, Georgia, USA

**Keywords:** lipopolysaccharide, lipid A, lipid synthesis, cellular regulation, outer membrane, LpxC, cell envelope

## Abstract

Gram-negative bacteria surround their inner membrane and cell wall with an asymmetric outer membrane which contains lipopolysaccharide (LPS) in its outer leaflet. In addition to serving as a potent permeability barrier, the LPS-rich outer membrane also contributes to the structural integrity of the cell envelope and, thus, the ability to synthesize LPS is essential in most Gram-negative bacteria. Although the cell must make enough LPS to support growth, its biosynthesis must be tightly regulated as overproduction of the acylated disaccharide domain of LPS, lipid A, is deleterious to bacterial viability. The committed enzyme of the lipid A biosynthetic pathway, LpxC, serves as a major regulatory node to control flux through the pathway and ensure that the cell has enough, but not too much, LPS. The regulation of LpxC in the model organism *Escherichia coli* has been the subject of immense study, which has led to a detailed understanding of the regulation of LPS biogenesis that has served as a paradigm for other organisms. Recent work, however, has revealed diverse mechanisms used by different species to regulate LpxC and highlights that regulation at alternative points in the LPS biosynthetic pathway have the potential to influence LPS production. Here, the discovery and subsequent molecular dissection of the varied strategies used to regulate LPS biosynthesis are reviewed as well as the physiological consequences of LPS dysregulation.

The Gram-negative cell envelope is a complex structure comprised of three concentric layers: an inner membrane (IM) composed of glycerophospholipids (GPLs), a cell wall made up of peptidoglycan (PG), and an asymmetric outer membrane (OM) with GPLs on the inner leaflet and an outer leaflet largely composed of lipopolysaccharide (LPS) (1). The permeability barrier imposed by this structure, due in large part to the presence of LPS in the OM, is a major driver of intrinsic antibiotic resistance observed in Gram-negative bacteria (2). LPS is an amphipathic molecule made up of three main structural domains: lipid A, core, and O-antigen. Lipid A is an acylated glucosamine-based disaccharide that anchors LPS in the lipid bilayer and is ligated to a series of outward-facing sugar residues that compose the core and O-antigen domains of LPS (3). Lipid A along with the first sugar(s) of the core, 3-deoxy-D-*manno*-oct-2-ulosonic acid (Kdo), collectively called Kdo-lipid A (Fig. 1*A*), is considered essential in most Gram-negative bacteria and the enzymes responsible for its production are highly conserved (4).

**Figure 1:**
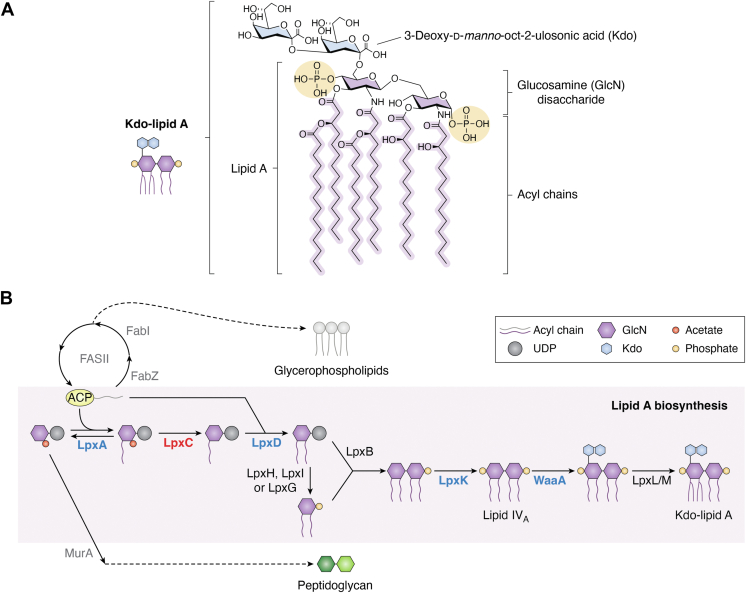
**Lipid A biosynthesis.***A*, chemical structure of Kdo-lipid A from *Escherichia coli*. *B*, depiction of the Kdo-lipid A biosynthesis pathway. LpxA, the first enzyme in the lipid A biosynthesis pathway, catalyzes the reversible acylation of UDP-GlcNAc using FAS II-derived β-hydroxyacyl-ACP molecules, metabolic precursors shared with the PG and GPL biosynthetic pathways, respectively. The LpxA product is deacetylated by the committed enzyme in the lipid A biosynthetic pathway, LpxC. Following LpxC catalysis, lipid A production is completed by a series of chemical reactions catalyzed by LpxD, LpxH/I/G, LpxB, LpxK, WaaA, LpxL, and LpxM. See main text for details. LpxC (highlighted in *red*) serves as the best characterized regulatory node in the LPS biosynthesis pathway, but other enzymes in the pathway (highlighted in *blue*) have also been implicated in controlling the production of LPS. Other relevant enzymes in the PG and FAS II synthesis pathways are presented in *gray*. Kdo, 3-deoxy-D-*manno*-oct-2-ulosonic acid; LPS, lipopolysaccharide; GPL, glycerophospholipid; PG, peptidoglycan; UDP-GlcNAc, uridine diphosphate-N-acetyl-glucosamine; ACP, acyl carrier protein; FAS II, type II fatty acid synthesis.

Kdo-lipid A is synthesized in the bacterial cytoplasm from a series of catalytic reactions that make up the Raetz pathway, named after Christian Raetz whose group pioneered the identification and characterization of the nine enzymes that make up the pathway (reviewed in (3, 5, 6) (Fig. 1*B*). The first enzyme in the Raetz pathway, LpxA, acylates uridine diphosphate-*N*-acetyl-glucosamine (UDP-GlcNAc) using a β-hydroxyacyl-acyl carrier protein (ACP) substrate made by the type II fatty acid synthesis pathway (FAS II) (7). The reaction catalyzed by LpxA is thermodynamically unfavorable, and it is the second enzyme in the pathway, LpxC, that catalyzes the first irreversible and committed step in lipid A biosynthesis (8, 9). LpxC is a deacetylase that removes the acetyl group from the GlcNAc moiety to allow for the addition of the next acyl group by LpxD and subsequent removal of UMP by LpxH, LpxI, or LpxG depending on the organism (Fig. 1*B*) (10, 11, 12, 13). LpxB then catalyzes the formation of a tetra-acylated disaccharide intermediate, which is phosphorylated by LpxK to produce lipid IV_A_ (14, 15) (Fig. 1*B*). Next, WaaA adds a number of Kdo residues that depends on the organism to form the first sugar(s) of the core, resulting in Kdo-lipid IV_A_ which is sequentially acylated by LpxL and LpxM to finally form Kdo-lipid A (16, 17, 18) (Fig. 1*B*).

The nine steps in the Raetz pathway as well as the subsequent addition of the core sugars are catalyzed in the cytoplasm or the cytoplasmic leaflet of the IM, but the resulting core-lipid A must ultimately be transported to the OM. Transport is achieved in two steps. In the first, core-lipid A is flipped from the cytoplasmic leaflet to the periplasmic leaflet of the IM by the flippase MsbA (reviewed in (19)). Once facing the periplasm, O-antigen can be ligated onto core-lipid A prior to its transport to the OM by the lipopolysaccharide transport (Lpt) system (reviewed in (20)).

The synthesis of lipid A and its subsequent assembly into the OM have long-been viewed as ideal targets for antibiotics, as it is essential and unique to Gram-negative bacteria. From numerous drug discovery endeavors, small molecules that inhibit the Raetz pathway enzymes and LPS transporters have been identified, a subset of which have progressed to clinical trials (21, 22). The physiological consequences associated with impaired LPS synthesis and assembly are vast, consistent with the essentiality of LPS. Intriguingly, overproduction of LPS results in similar growth inhibition, with the physiological consequences discussed below. A detailed understanding of the molecular mechanisms used by bacteria to increase or reduce the production of LPS therefore has the potential to directly inform therapeutic development.

## Effects of misregulated lipid A biosynthesis

**Figure 2:**
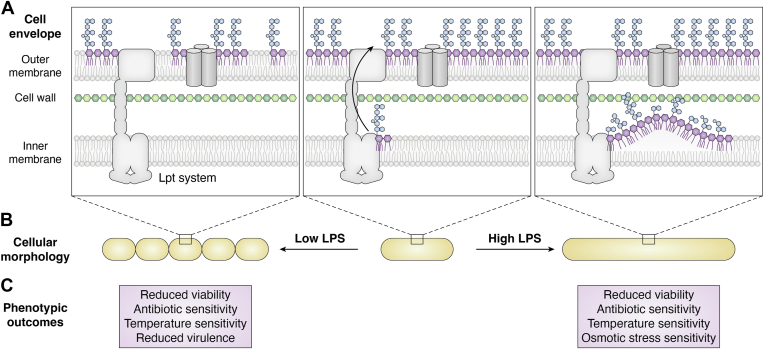
**Phenotypic consequences of misregulated production of lipid***A*, summary of phenotypes associated with Gram-negative bacterial strains that exhibit reduced (*left*), wild-type (*middle*), or elevated (*right*) production of LPS. *A*, graphic representation of the cell envelope under each condition. Lipid A is depicted in *purple* and the core and O-antigen domains of LPS are depicted in *blue*. When LPS production is impaired, phospholipids accumulate in the outer leaflet of the OM, disrupting the permeability barrier. In WT cells under standard laboratory conditions, the Lpt system readily transports nascent LPS to the OM, leading to low levels of LPS in the IM and robust OM function. When LPS production is heightened to the point where it exceeds the transport capacity of the Lpt system, LPS intermediates accumulate in the inner membrane and form periplasmic membrane structures. *B*, changes to *E. coli* cellular morphology observed upon altered LPS biosynthesis. When LPS production is reduced, chains of shortened cells are observed. When LPS production is increased, cellular filamentation is elevated. *C*, select phenotypic outcomes associated with reduced and elevated LPS biosynthesis. LPS, lipopolysaccharide; IM, inner membrane; OM, outer membrane; Lpt, lipopolysaccharide transport.

The ability of Gram-negative bacteria to control lipid A biosynthesis is critical for viability. In most Gram-negative organisms, LPS is essential and thus too little LPS can directly impair growth and viability (23, 24). Even slight decreases in LPS production can have substantial effects on membrane permeability and function. Indeed, mutations that reduce LPS biosynthesis have been reported to result in cellular chaining (25, 26, 27), reduced virulence (28), and increased sensitivity to both antibiotics and high temperature (29, 30, 31, 32) (Fig. 2). In addition, it is becoming increasingly apparent that LPS plays a significant role in the structural integrity of the cell envelope, a function that has historically been attributed to the cell wall (33, 34, 35). As such, there is substantial selective pressure to ensure that cells produce enough LPS to support proper OM function.

Hyperproduction of LPS is similarly detrimental to cellular viability for a variety of reasons. Lipid A biosynthesis relies on acyl-ACP and UDP-GlcNAc precursors, which are essential for GPL and PG biosynthesis, respectively (36, 37, 38) (Fig. 1*B*). Thus, elevated synthesis of LPS has the potential to indirectly affect GPL and PG production due to competition for shared precursors. In addition, once LPS levels exceed the capacity allowed by the OM and the Lpt transport system, LPS accumulates in the IM, which results in the formation of periplasmic membrane structures (39, 40) (Fig. 2). This aberrant accumulation of LPS in the IM appears to be toxic, as some of the growth defects associated with *Escherichia coli* mutants that hyperproduce LPS can be suppressed by deleting a protein that tethers the OM to the cell wall, *lpp*, thereby elevating OM vesiculation to promote the loss of OM-embedded LPS and make space for nascent LPS (31, 41). Furthermore, there is evidence that detergent-like LPS intermediates that accumulate upon hyperactivation of early stages in the Raetz pathway can be toxic, as depletion of the later-stage enzymes LpxH and LpxK resulted in growth defects that could be suppressed by inhibition of LpxA or LpxC in *Acinetobacter baumannii*, one of the few known organisms in which LPS is not essential (42, 43, 44). In addition to reduced viability, mutant strains that overproduce lipid A exhibit cellular filamentation, incomplete LPS maturation (40, 41), antibiotic sensitivity (45, 46), osmotic stress sensitivity (46), and temperature sensitivity (41, 46) (Fig. 2). Thus, there is also substantial selective pressure to prevent cells from producing too much LPS.

To ensure that enough, but not too much, LPS is synthesized, Gram-negative bacteria tightly regulate the production of lipid A. The regulatory strategies that govern lipid A biosynthesis are of immense interest due not only to their fascinating biological implications, but also their connection to bacterial viability and OM permeability, which are directly relevant to therapeutic development. Although the physiological impacts of misregulated LPS biosynthesis are likely to be conserved, recent work has highlighted that the molecular strategies used by bacteria to control lipid A production are not. Instead, diverse bacteria have evolved unique systems to modulate the synthesis of this critical cell envelope component. Work over the past several decades has revealed a variety of molecular mechanisms that regulate the production of LPS, often at the level of the committed enzyme in the pathway, LpxC.

## Regulation of LpxC in *E. coli*

Many of the early studies of lipid A biosynthesis focused on *E. coli* and, as such, it is the organism in which the regulation of lipid A biosynthesis has been best described. In particular, LpxC in *E. coli* (^Ec^LpxC) has emerged as a critical point of regulation to control flux through the Raetz pathway and the molecular characterization of the factors that influence its activity has yielded a detailed model of how LPS synthesis is regulated in this organism. This work has been formative to the field and has provided a basis for understanding the interconnected pathways that control cell envelope biosynthesis.

### ^Ec^LpxC is proteolytically regulated by FtsH

The first evidence of lipid A biosynthesis regulation was reported in the same study that identified LpxC as the committed enzyme in the pathway. Specifically, Anderson et al found that LpxC activity was raised 5- to 10-fold in temperature-sensitive LpxA mutants grown in the nonpermissive condition (8). However, the manner by which LpxC was regulated did not become clear until a subsequent study that showed LpxC activity increased post transcriptionally under conditions that reduced lipid A production (47). These studies suggested the level of lipid A, or some biosynthetic intermediate, was sensed by an unknown mechanism and used to inform the rate of either LpxC translation or proteolysis. The specifics of this newfound regulation, however, were unclear.

FtsH was identified shortly thereafter as a protease that targets LpxC. FtsH is a hexameric, ATP-dependent, and membrane-embedded protease that is broadly conserved among bacteria (48). Early attempts to characterize FtsH in *E. coli* found it to be essential, but a strain harboring a catalytically inactive variant of FtsH was able to be isolated through the acquisition of a secondary suppressor mutation termed *sfhC21* (for suppressor of *ftsH*) (49, 50, 51). Ogura et al subsequently mapped the *sfhC21* mutation to a missense allele in *fabZ*, encoding the hypermorphic FabZ (L85P) variant (39). FabZ is situated at a critical point in the FAS II system: it catalyzes the dehydration of β-hydroxyacyl-ACPs to continue acyl chain elongation (52). Although it is one of two β-hydroxyacyl-ACP hydratases encoded by *E. coli*, it is largely responsible for the elongation of saturated long chain substrates, including the ^Ec^LpxA substrate *R-*3-hydroxymyristoyl-ACP (53). Thus, once acyl chains have reached the appropriate length, FabZ competes with ^Ec^LpxA for their common substrate. As such, the level of FabZ activity has the potential to directly impact lipid A production through impacting the abundance of a shared precursor. Indeed, a hypomorphic FabZ variant had been previously shown to suppress a temperature-sensitive *lpxA* mutant, likely through elevating the availability of *R*-3-hydroxymyristoyl-ACP (52). Thus, Ogura et al reasoned that the essential function of FtsH may be through modulation of membrane lipid biosynthesis. Subsequent analysis of GPL and LPS production revealed that, although GPL levels were largely unchanged, LPS accumulated approximately 50% more when FtsH was inactivated compared to the permissive conditions (39). Furthermore, the accumulation of LPS in the absence of FtsH was suppressed by the *sfhC21* allele, suggesting that the hyperproduction of LPS was related to the essential function of FtsH (39).

With the knowledge that FtsH modulates LPS biosynthesis in hand, Ogura et al wondered if FtsH was the post transcriptional regulator of LpxC that had been previously reported. Indeed, they found that the specific activity of LpxC increased in *ftsH* mutant lysates and observed a concomitant accumulation of LpxC protein (39). Furthermore, *in vitro* biochemical characterization showed that FtsH was able to directly degrade LpxC, confirming FtsH as the first example of a direct regulator of lipid A biosynthesis (39). This seminal study not only explained how ^Ec^LpxC was specifically regulated but also emphasized several themes in the regulation of cell envelope synthesis that have greatly influenced the field. Namely, hyperaccumulation of LPS is detrimental to cells and balancing LPS biosynthesis with the production of other cell envelope components is critical.

### FtsH-mediated proteolysis of ^Ec^LpxC is controlled by LapB and YejM

FtsH is responsible for the degradation of numerous protein substrates in addition to LpxC, including the heat-shock sigma factor σ^32^, the lambda regulator cII, and diverse membrane proteins (54, 55, 56, 57, 58, 59). It seemed clear that FtsH was able to discriminate among its substrates in response to specific signals, however, as altered LPS biosynthesis specifically impacted the ability of FtsH to degrade ^Ec^LpxC and not another proteolytic target, σ^32^ (39). It was subsequently shown that ^Ec^LpxC contains a C-terminal degron sequence composed of six nonpolar residues (LAXXXXXAVLA) that is critical for its ability to be recognized by FtsH (60, 61). Notably, this degron sequence is distinct from other FtsH substrates (48, 60), but it was unclear how FtsH recognized such diverse substrates and specifically singled out LpxC for degradation under conditions in which LPS production was high.

In 2014, several groups independently reported the involvement of a heat shock responsive IM-protein LapB (lipopolysaccharide assembly protein B; formerly called YciM) in cell envelope integrity (41, 45, 46). Specifically, it was shown that *lapB* mutation was only tolerated in a subset of *E. coli* strains and that those strains in which *lapB* was mutated exhibited hypersensitivity to antibiotics, elevated cell envelope stress response pathways, and filamentous cellular morphology similar to that observed upon overexpression of ^Ec^LpxC (41, 45, 46, 61). Two of these initial studies took advantage of the dramatic phenotypes associated with *lapB* deletion and isolated mutants that restored growth under nonpermissive conditions, many of which mapped to the ^*Ec*^*lpxC* gene (41, 45). Furthermore, ^Ec^LpxC levels increased in the absence of LapB, resulting in an accumulation of LPS (41, 45). Importantly, *lapB* deletion did not affect the stability of σ^32^, another FtsH substrate, leading one group to propose that LapB might serve as an adapter that facilitated regulated proteolysis of ^Ec^LpxC by FtsH (45). In support of this model, LapB was shown to copurify with FtsH (41).

The role of LapB as an adaptor protein was later confirmed by biochemical studies in which LapB addition dramatically elevated the rate in which FtsH degraded ^Ec^LpxC (62). Detailed enzymatic characterization revealed that the increased FtsH-mediated degradation of ^Ec^LpxC observed in the presence of LapB was likely a result of enhanced binding affinity for ^Ec^LpxC (62). In further support of LapB serving as an adaptor, it was shown to interact directly with both FtsH (through its transmembrane helix) and ^Ec^LpxC (through tetratricopeptide repeats in its cytosolic domain) (62). More recently, structural analysis of LapB-^Ec^LpxC interaction has revealed that LapB makes extensive contacts with the N terminal and central regions of the ^Ec^LpxC degron, bringing the C terminal tail in close proximity to the membrane where it would be readily accessible to the FtsH active site (63). Intriguingly, LapB also makes extensive contacts near the hydrophobic tunnel of ^Ec^LpxC that accommodates substrate binding and, consistent with this, LapB binding is sufficient to reduce ^Ec^LpxC activity by approximately 80% *in vitro* (63). Thus, LapB appears to regulate ^Ec^LpxC at two levels: first, LapB binds to ^Ec^LpxC to immediately reduce its activity and second, it directs ^Ec^LpxC to FtsH for proteolysis (63). Why LapB might inhibit ^Ec^LpxC *via* two mechanisms and whether both are physiologically relevant under all conditions is unclear. Intriguingly, LapB expression is elevated after heat shock, a condition that is associated with an increase in unfolded proteins which have been suggested to be targeted by FtsH (59). Perhaps heat shock or other proteotoxic conditions result in increased competition between ^Ec^LpxC and unfolded proteins for proteolysis by FtsH, thereby increasing the need for direct inhibition of ^Ec^LpxC by LapB. Future work to carefully disentangle the functions of LapB as an adaptor and inhibitor are needed to clarify its precise role in the regulation of LpxC.

The discovery of LapB provided an explanation for how FtsH could specifically degrade ^Ec^LpxC under certain conditions without affecting its other proteolytic targets. How the status of LPS biosynthesis was sensed and used to inform FtsH-mediated proteolysis of ^Ec^LpxC, however, was still unknown. Recently, it has become clear that the membrane protein YejM (called PbgA in *Salmonella*) is capable of both binding to periplasmic LPS and acting as an antiadaptor of LapB, thus serving to sense LPS levels and modulate ^Ec^LpxC degradation accordingly. As early as 1997, a mutant strain termed LH530 was reported to have phenotypes characteristic of altered OM biogenesis including sensitivity to antibiotics, periplasmic leakage, and reduced LPS biosynthesis, but at the time the location of the mutation that caused these phenotypes was unknown (64, 65). Over a decade later, the LH530 phenotypes were shown to be a result of a nonsense mutation in *yejM* that significantly truncated the protein (66). Although LH530 was viable when propagated at low temperature, complete deletion of *yejM* was not tolerated under any conditions tested, indicating that YejM was essential for viability (66). The essential function of YejM remained unclear until several groups independently reported a connection between YejM and ^Ec^LpxC in 2019 to 2020.

To better understand why YejM was essential, one group set out to biochemically characterize YejM and found that it copurified with LapB (67). Concurrently, several other groups tackled the question by isolating genetic suppressors of *yejM* mutants and all identified mutations in ^*Ec*^*lpxC, lapB,* and/or *ftsH* (27, 28, 31, 32). The biochemical and genetic interactions between YejM and ^Ec^LpxC/LapB/FtsH were all predicted to impact ^Ec^LpxC specific activity, indicating that YejM mutation might alter ^Ec^LpxC levels. Indeed, ^Ec^LpxC abundance was dramatically reduced in *yejM* mutants, resulting in decreased production of LPS (27, 30, 31, 32, 67). Further, structural characterization of YejM showed that it contained an LPS binding pocket in its periplasmic surface, providing a means to sense the status of LPS biosynthesis (67).

**Figure 3:**
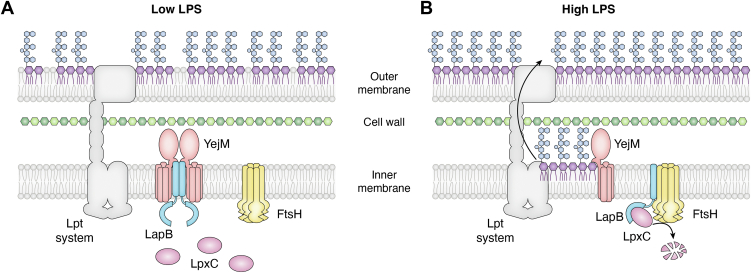
**Regulation of LpxC in *Escherichia coli*.***A*, when LPS levels are low, YejM binds to LapB, preventing it from promoting FtsH-mediated proteolysis of LpxC. *B*, under conditions in which LPS levels are high, YejM binds to LPS molecules that accumulate in the outer leaflet of the IM, allowing LapB to bind LpxC and direct it for proteolysis by FtsH. Lipid A is depicted in *purple* and the core and O-antigen domains of LPS are depicted in *blue*. LPS, lipopolysaccharide; IM, inner membrane; LapB, lipopolysaccharide assembly protein B.

Together, these studies supported a model in which YejM inhibits LapB-FtsH mediated proteolysis of ^Ec^LpxC unless LPS accumulates in the periplasmic leaflet of the cytoplasmic membrane (27, 30, 31, 67). Consistent with this, subsequent biochemical and structural analyses showed that YejM directly interacted with LapB, that the LPS and LapB binding sites of YejM overlapped, and that the addition of LPS to YejM reduced its capacity to interact with LapB (62). Further, YejM sequestered the same region of LapB used to interact with FtsH (62). Thus, a series of partner switching events elegantly explains the ability of ^Ec^LpxC levels to respond to changes in LPS production (Fig. 3). Under conditions in which LPS levels are relatively low, YejM sequesters LapB, preventing FtsH-mediated proteolysis of ^Ec^LpxC (Fig. 3*A*). In contrast, when LPS levels are relatively high, LPS intermediates accumulate in the periplasmic leaflet of the IM as they await transport and bind to YejM (Fig. 3*B*). When YejM is bound to LPS, LapB is freed to inhibit ^Ec^LpxC and subsequently direct it to FtsH for proteolysis to reduce lipid A biosynthesis (Fig. 3*B*).

### Alternative signals modulating ^Ec^LpxC abundance

Although the picture of how LpxC is regulated in *E. coli* is becoming clearer, there are many observations that suggest additional complexity may exist in the control of lipid A biosynthesis. Stimuli that control ^Ec^LpxC levels have been reported that, at first glance, are not accounted for with our current understanding of the YejM-LapB-FtsH system. In particular, several studies have highlighted the influence of perturbations to OM asymmetry on ^Ec^LpxC regulation. In *E. coli*, OM asymmetry is maintained through the action of multiple systems, including the phospholipase PldA that degrades GPLs localized in the outer leaflet of the OM to produce lysophospholipids and fatty acids (68, 69). Under conditions in which GPLs accumulate in the outer leaflet of the OM, PldA activity has been shown to stabilize LpxC and increase LPS biosynthesis (70). This function of PldA was dependent on the presence of FadD, which imports exogenous fatty acids and simultaneously activates them with coenzyme A (CoA) to produce cytosolic acyl-CoA species (70, 71, 72). Similarly, strains with mutations in *yhcB*, which encodes a transmembrane protein that has been shown to copurify with LapB, exhibit altered ^Ec^LpxC abundance in addition to many pleiotropic phenotypes often associated with defects in cell envelope integrity (73, 74). However, a recent report indicated that the effects of YhcB on ^Ec^LpxC are likely indirect due to its modulation of fatty acid biosynthesis to alter GPL production (75). In the absence of YhcB, the ratio of GPLs to lipid A elevates, presumably causing an accumulation of mislocalized GPLs in the outer leaflet of the OM that serve as substrates for PldA to indirectly impact ^Ec^LpxC levels (75). These results support an attractive model whereby an increase in mislocalized GPLs that result from reduced LPS biosynthesis, increased GPL biosynthesis, or other OM perturbations may be sensed to inform the cytosolic production of lipid A.

The mechanism by which acyl-CoA species might influence ^Ec^LpxC is unknown but addition of exogenous palmitate to growing *E. coli* cultures has similarly been shown to stabilize ^Ec^LpxC (76). It is notable that palmitoyl-CoA has been reported to inhibit the activity of the essential FAS II enzyme FabI, and a reduction in FabI activity has, in turn, been reported to increase ^Ec^LpxC levels, perhaps explaining why ^Ec^LpxC might be sensitive to palmitoyl-CoA (39, 77). Alternatively, long-chain acyl-CoA species derived from exogenous fatty acids have been recently suggested to indirectly increase long-chain acyl-ACP species, which reduces the production of nascent acyl-ACPs through the inhibition of acetyl-CoA carboxylase (78, 79). Thus, the addition of exogenous long-chain fatty acids to *E. coli* led to a rapid and dramatic remodeling of the acyl-ACP chemical landscape, including a reduction in the LpxA substrate *R*-3-hydroxymyristoyl-ACP (78). Perhaps consistent with this, exogenous addition of oleate to growing cultures has been shown to reduce LPS levels (80). As such, it is possible that the stabilization of ^Ec^LpxC observed upon addition of exogenous fatty acids is a result of reduced LPS produced as a function of decreased substrate availability. However, future work is needed to clarify if PldA-mediated phospholipid degradation mirrors the effects of exogenous fatty acid addition on *de novo* fatty acid biosynthesis and how these alterations directly impact ^Ec^LpxC regulation.

^Ec^LpxC turnover has also been shown to be responsive to growth rate: under fast-growth conditions ^Ec^LpxC is relatively stable (with a half-life up to 2 h), but in conditions in which growth is slow, ^Ec^LpxC is rapidly turned over (with a half-life as low as 4 min) (81). This effect appears to be specific to ^Ec^LpxC, as the opposite is observed for the other FtsH substrate CII, which is rapidly degraded under fast-growth conditions but has an extended half-life when cells are grown at lower temperatures (82). The influence of growth rate on ^Ec^LpxC stability was further shown to be mediated at least in part by (p)ppGpp, the secondary messenger that controls the stringent response to alter cellular physiology under various stressful conditions such as fatty acid starvation (81, 83). How (p)ppGpp impacts ^Ec^LpxC stability is unknown, but it is likely to be indirect as binding studies did not support a direct interaction between the secondary messenger and either ^Ec^LpxC or FtsH (81, 84). Intriguingly, activation of the stringent response impairs GPL biosynthesis at multiple points, including by reducing the activity of FabZ (85, 86). Thus, an elevation in (p)ppGpp levels may reduce the GPL:LPS ratio, leading to a buildup of LPS in the outer leaflet of the IM that is sensed by YejM to derepress LapB/FtsH-mediated ^Ec^LpxC degradation. Alternatively, it has been reported LapB production elevates during stationary phase in *Salmonella enterica* serovar Typhimurium, which leads to an increase in LpxC turnover compared to exponential phase (87). It is therefore possible that changes in protein expression, rather than GPL biosynthesis, could mediate altered ^Ec^LpxC regulation under different growth conditions.

Finally, ^Ec^LpxC levels have been shown to be reduced when the small RNA GcvB is overexpressed (88). GcvB is reported to regulate over 50 mRNAs in *E. coli*, which are generally involved in amino acid transport and metabolism (89). Notably, the mRNAs that encode LpxC and its recognized regulators are not among the known targets of GcvB. Deletion of *gcvB* increases sensitivity to the LpxC inhibitor CHIR-090, perhaps consistent with a role in controlling lipid A biosynthesis (88). Alternatively, the increased expression of permeases and importers that occurs upon deletion of *gcvB* may allow increased uptake of CHIR-090, thereby indirectly affecting sensitivity as has been suggested for aminoglycosides (90). Thus, many questions remain as to how GcvB impacts ^Ec^LpxC abundance and CHIR-090 sensitivity, and further investigation is needed to clarify its involvement in the regulation of lipid A biosynthesis.

### Relationship to other elements of cell envelope assembly

Several lines of evidence suggest that the YejM-LapB-FtsH system is integrated into a larger network of cell envelope assembly factors. In particular, YejM has been shown to copurify with the GPL biosynthesis enzyme PlsY, and LapB has also been shown to copurify with numerous other proteins involved in membrane biogenesis, including LpxA, LpxD, FabZ, YhcB, components of the Lpt system, and the LPS-core assembly protein WaaC (41, 67, 91). LapA (formerly YciS) is encoded in a dicistron with LapB and was also shown to copurify with LapB (41). Although phenotypes associated with *lapA* deletion are minor compared with the mutations in *lapB*, *lapA* mutants have growth defects on MacConkey agar at high temperatures and also exhibit a slightly altered profile of lipid A species (41). Consistent with a possible role in influencing FtsH-mediated proteolysis of ^Ec^LpxC, a nonsense mutation in *lapA* was shown to suppress *yejM* deletion (27). However, it is unclear how LapA might influence LapB, and it remains to be validated if its ability to interact with LapB is the basis for the phenotypes associated with *lapA* mutations. The biological significance of these varied interactions is largely unknown, but it is appealing to speculate that YejM and LapB might serve as critical nodes to orchestrate the coordinated synthesis and assembly of the cell envelope.

Although it is now clear that YejM contributes directly to the control of LPS biosynthesis, the possibility that it has a role in other functions of the cell envelope has been reported on several occasions. YejM is composed of a transmembrane domain connected to a globular periplasmic domain by an interfacial domain (67, 92) and exhibits strong homology to enzymes involved in cell envelope modifications, including the lipoteichoic acid synthase gene LtaS and the LPS modifying enzyme EptA (93). Both LtaS and EptA are metalloenzymes that require Mn^2+^ and Zn^2+^, respectively, to be catalytically active (94, 95), but the residues that coordinate those cations are not conserved in YejM (67). Instead, the periplasmic domain of YejM has been reported to be a Mg^2+^-dependent phosphatase (93), although the native substrate has not been identified and phenotypes associated with YejM variants that ablate Mg^2+^ binding have not been reported. As such, the biological relevance of YejM's phosphatase activity is unclear. In addition, early reports found that *S.* Typhimurium and *Shigella flexneri* strains in which *yejM* homologs had been mutated exhibited reduced cardiolipin in their OM, leading to a model that YejM might function as a transporter for cardiolipin between the IM and OM (96, 97). Mutation of two consecutive arginine residues in a putative cardiolipin binding site in the interfacial domain of the *S.* Typhimurium YejM homolog PbgA resulted in growth defects and increased OM permeability (92, 97). However, it is now clear that the interfacial domain of YejM predominantly binds to LPS and utilizes the same arginine residues that had been previously implicated in cardiolipin transport to the OM (67). Thus, it is possible that the impacts of YejM/PbgA on cardiolipin transport may be indirect through its influence on LPS biosynthesis and additional work is necessary to disentangle these phenotypes.

## Regulation of LpxC in *Pseudomonas aeruginosa*

Although FtsH is highly conserved within the bacterial domain, a 2011 study indicated that its ability to regulate LpxC was largely restricted to Enterobacteriaceae (98). In particular, it was observed that the C terminal region of LpxC from *P. aeruginosa* (^Pa^LpxC) among other organisms diverged significantly from the degron sequence identified in ^Ec^LpxC, suggesting that these organisms may not control LpxC through FtsH-mediated proteolysis (98). Indeed, FtsH is not essential in *P*. *aeruginosa* where it was later shown to play an important role in proteostasis in response to stress as opposed to direct regulation of cell envelope synthesis (99). In addition, experiments in which ^Pa^LpxC degradation was monitored over time suggested that it was likely not subject to proteolysis at all (98). Nevertheless, ^Pa^LpxC was still proposed to be a critical regulatory node as heterologous overexpression of ^Ec^LpxC in *P. aeruginosa* resulted in a dramatic reduction in viability, but overexpression of ^Pa^LpxC had no obvious phenotype (98). Thus, the authors predicted that the activity of the ^Pa^LpxC was held in check by some regulatory strategy that did not affect ^Ec^LpxC, but it was not clear what factors were involved (98).

Over a decade later it was revealed that ^Pa^LpxC is regulated by a wholly unique mechanism: it is activated by direct binding to the committed enzyme in the competing PG biosynthetic pathway, MurA (100). The interaction between ^Pa^LpxC and MurA was initially revealed in a coaffinity purification assay to identify interactors of ^Pa^LpxC and subsequently biochemically and physiologically characterized (100). Biochemical characterization of this interaction showed that MurA and ^Pa^LpxC likely interacted at a 1:1 ratio and that MurA was sufficient to stimulate ^Pa^LpxC activity *in vitro* (100). Furthermore, this study showed that expression of a catalytically dead MurA variant (C117S) in otherwise WT *P. aeruginosa* resulted in both the accumulation of LPS as well as the ^Pa^LpxC product (UDP-3-O-(*R*-3-hydroxydecanoyl)-glucosamine) (100). The toxicity of the MurA (C117S) variant depended on its ability to interact with ^Pa^LpxC, indicating that its physiological effects were due to constitutive activation of LPS biosynthesis while being unable to contribute to its canonical function in PG biosynthesis (100). In addition to being sufficient to activate ^Pa^LpxC, MurA was also shown to be necessary for production of LPS. Indeed, depletion of MurA in *P. aeruginosa* resulted in reduced LPS levels and changes to cellular morphology similar to that observed upon simultaneous inhibition of LPS and PG biosynthesis with antibiotics that target their respective pathways (100). Perhaps more importantly, a variant of MurA that was still enzymatically active but could not interact with ^Pa^LpxC was unable to support viability, indicating that MurA's ability to stimulate LPS biosynthesis through activation of ^Pa^LpxC is essential in *P. aeruginosa* (100).

LpxC and MurA are both highly conserved among proteobacteria, making it challenging to identify other organisms in which MurA-mediated activation of LpxC may be at play. To predict other species that encode MurA/LpxC pairs that may interact, covariation analysis of residues predicted by Alphafold modeling to interact in the *P. aeruginosa* LpxC-MurA complex was performed (100). This experiment identified diverse gammaproteobacteria as well as a subset of alphaproteobacteria in which LpxC and MurA were likely to interact, and the accuracy of the predictions was confirmed through subsequent biochemical characterization of a subset of LpxC/MurA cognate pairs (100). Thus, it is likely that the ability of MurA to stimulate LPS biosynthesis is observed beyond *P. aeruginosa* and its close relatives, but future work is needed to characterize the physiological effects of LpxC and MurA interaction in those species.

**Figure 4:**
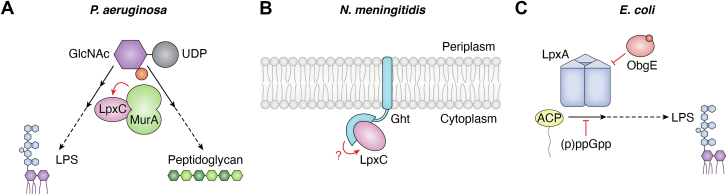
**Alternative strategies that control lipid A biosynthesis.** Graphic depictions of a subset of systems proposed to regulate the production of lipid A in diverse Gram-negative bacteria. *Red arrows* and *T-bars* indicate suggested regulation by activation and inhibition, respectively, whereas *black arrows* indicate enzymatic reactions. Lipid A is depicted in *purple* and the core and O-antigen domains of LPS are depicted in *blue*. *A*, in *P. aeruginosa*, LpxC is activated by direct binding to MurA, the committed enzyme in peptidoglycan biosynthesis which shares the metabolic precursor UDP-GlcNAc with LPS biosynthesis. *B*, in *N. meningitidis*, the LapB homolog Ght promotes LPS biosynthesis by binding to LpxC, although the mechanism of regulation is unclear. *C*, in *Escherichia coli*, the first enzyme in lipid A biosynthesis, LpxA, has been reported to be directly inhibited by the essential GTPase ObgE as well as indirectly inhibited by the stringent response secondary messenger (p)ppG.pp. UDP-GlcNAc, uridine diphosphate-N-acetyl-glucosamine; LapB, lipopolysaccharide assembly protein B.

Notably, MurA-mediated activation of LpxC was the first example of a committed enzyme in one pathway directly regulating the committed enzyme in a competing biosynthetic pathway. It was proposed that this mechanism of regulation was critical to ensure the proper balance between the LPS and PG biosynthetic pathways: LpxC is only active when bound to MurA, thus reducing the risk of runaway flux through the LPS biosynthetic pathway (Fig. 4*A*). Intriguingly this regulatory strategy emphasizes the importance of controlling utilization of the metabolic precursor shared between LPS and PG biosynthesis, UDP-GlcNAc. This is in contrast to the YejM-LapB-FtsH module which appears to fine-tune the balance of LPS and GPL biosynthesis by controlling the utilization of β-hydroxyacyl-ACP molecules. It is not clear why some organisms might favor balancing one pair of pathways over the other or if the two systems are mutually exclusive. However, it is enticing to think that diverse metabolic niches require different inputs to control flux through the cell envelope biosynthetic pathways. For example, *P. aeruginosa* preferentially consumes tricarboxylic acids as a carbon source as opposed to sugars consumed through glycolysis (101). As such, it seems plausible that UDP-GlcNAc, which is derived from the glycolytic intermediate fructose-6-P, may be more limiting in *P. aeruginosa* than in *E. coli* where glucose is a preferred carbon source (102). Further characterization of the metabolic consequences of misregulated LPS biosynthesis in diverse bacteria will be critical to investigate this model. Although MurA appears to be both necessary and sufficient to stimulate ^Pa^LpxC activity, it is unclear whether it does so constitutively or if its ability to activate ^Pa^LpxC could be modulated in response to certain conditions like that observed for the regulation imparted by YejM-LapB-FtsH. Furthermore, the possibility that additional regulators of ^Pa^LpxC or other *P. aeruginosa* lipid A biosynthetic enzymes might exist cannot be excluded. Thus, despite the recent progress, much remains unknown about how the lipid A biosynthesis pathway is regulated in *P. aeruginosa*.

## Regulation of LpxC in other organisms

Alternative mechanisms to control LpxC activity have been reported in diverse bacteria, but the details of these systems are still emerging. In alphaproteobacteria such as *Agrobacterium tumefaciens* and *Rhodobacter capsulatus*, LpxC lacks the C-terminal degron that is indicative of FtsH targeting (98). Nevertheless, when heterologously expressed in *E. coli*, both *A. tumefaciences* and *R. capsulatus* LpxC was degraded (98). This turnover of LpxC was shown to be due to the Lon protease, suggesting that Lon might serve as a regulator of LpxC in these bacteria (98). This work has yet to be extended into the native organisms, however, and it is unclear whether the *A. tumefaciens* and *R. capsulatus* Lon proteases are capable of recognizing their cognate LpxC and if the interaction is truly regulatory or nonspecific targeting due to LpxC misfolding.

LpxC has more recently been reported to be regulated at the level of transcription by two component systems in two other alphaproteobacterial species, *Rhodobacter sphaeroides* and *Caulobacter crescentus* (103, 104). The NtrYX two component system has been implicated in controlling numerous pleiotropic phenotypes, including adaptation to low O_2_, pathogen intracellular survival, and nitrogen fixation (105, 106, 107). Recent work, however, suggests that, at least in a subset of alphaproteobacteria, NtrYX can also impact cell envelope composition (108, 109). In both *R. sphaeroides* and *C. crescentus*, NtrYX inhibits the transcription of LpxC (103, 110). In *R. sphaeroides*, an *ntrYX* mutant exhibited approximately nine-fold elevated *lpxC* transcript abundance and two-fold accumulation of LPS compared with the WT (103). In *C. crescentus*, an additional layer of control is imposed by the ChvGI two component system, which activates *lpxC* transcription (104). However, both the NtrYX and ChvGI systems have regulons composed of dozens of genes that are coregulated with LpxC, so these systems do not specifically modulate LpxC activity like those described above.

LpxC in betaproteobacteria such as *Neisseria meningitidis* similarly lack a C-terminal degron. Nevertheless, LapB homologs are observed in these taxa. In one study, the *N. meningitidis* LapB homolog Ght was suggested to regulate LpxC through an FtsH-independent mechanism by localizing LpxC to the IM to promote LPS biosynthesis (111). Indeed, *ght* mutants exhibited reduced LPS biosynthesis and growth defects that could be suppressed through the elevated expression of LpxC (111). LpxC was further shown to localize to the IM of *N. meningitidis* in a Ght-dependent manner (111) (Fig. 4*B*). Why localization of LpxC to the IM would be beneficial and how that promotes LPS biosynthesis is unknown. Given the recent report that LapB in *E. coli* has the ability to alter LpxC activity through direct binding, however, it seems possible that Ght might directly influence LpxC activity in addition to its subcellular localization (63). Thus, Ght may in fact serve as an activator of LpxC, but future work is needed to clarify its function.

## Regulation of alternative points in lipid A biosynthesis

Although the regulation of LxpC is best described, there is mounting evidence that the modulation of other enzymes within the lipid A biosynthesis pathway might control the production of LPS. In particular, the first enzyme in the pathway, LpxA, is emerging as an alternative point of regulation. In *E. coli*, LpxA activity has recently been shown to be altered by several factors, including (p)ppGpp, the RNase H proteins RnhA and RnhB, and the essential GTPase ObgE (84, 112) (Fig. 4*C*). LpxA activity was consistently reduced in the presence of (p)ppGpp *in vitro*, although the effects of (p)ppGpp appear to be indirect through binding to the LpxA substrate *R*-3-hydroxyacyl-ACP (84) (Fig. 4*C*). Overexpression of LpxA in a mutant unable to make (p)ppGpp resulted in elevated OM vesiculation and cellular filamentation, but it is unclear if these effects are due to an increase in LpxA activity in the absence of (p)ppGpp or due to additive effects associated with both genetic perturbations (84). LpxA has also been shown to be inhibited by a dominant-negative variant of ObgE (ObgE∗), an essential GTPase best known for its role in regulating ribosome assembly (112). The ObgE∗ variant is toxic when expressed in otherwise WT cells and results in phenotypes associated with OM defects in *E. coli* (112). Intriguingly, the ObgE∗ phenotypes could be suppressed by mutations in LpxA and further biochemical characterization showed that ObgE∗ and, to a lesser extent, the WT ObgE protein (ObgE^WT^) were able to interact directly with LpxA (112). Although the ability of ObgE^WT^ to target LpxA was muted compared to the ObgE∗ variant, overexpression of ObgE^WT^ had slightly elevated resistance to an antibiotic that targets LpxC and CRISPRi-mediated depletion of ObgE resulted in elevated LPS content, supporting a model by which ObgE inhibits LpxA activity *in vivo* (112) (Fig. 4*C*). In contrast, RnhA and RnhB have been shown to stimulate LpxA activity *in vitro,* although the conditions in which this regulatory mechanism might be relevant are unclear (84).

LpxA regulators in other species have also been reported. In the gammaproteobacterium *Cronobacter sakazakii*, LpxA was found to interact with LabP. Consistent with LabP serving as an activator of LpxA, *labP* mutants had reduced core-lipid A and increased GPL levels (113). Furthermore, addition of purified LabP was sufficient to stimulate LpxA activity *in vitro*, suggesting that it directly regulated LpxA (113). LabP is annotated as an acetyltransferase, but it exhibits substantial homology to LpxA (26.7% identity according to clustal omega v 2.1) (114). LpxA functions as a trimer (115), raising the possibility that a heterooligomer between LpxA and LabP may exist under certain conditions in *C. sakazakii*. In *Francisella tularensis*, LpxA interacts with another protein, RipA, that is required for replication within host cells (116). Perhaps counterintuitively, although *ripA* mutants have reduced LpxA levels, RipA was suggested to inhibit LpxA activity due to the ability of hypomorphic LpxA variants to suppress *ripA* mutant phenotypes (116). However, neither mutation of *ripA* nor the expression of the hypomorphic LpxA variants had a measurable impact on membrane composition (116). Thus, although it is clear that LpxA has the capacity to interact with a variety of protein partners in diverse organisms, further work is needed to understand the biological significance of these interactions and how they contribute to the regulated production of lipid A.

Like LpxC, two other enzymes in the lipid A biosynthesis pathway, LpxD and WaaA, have been reported to be subject to FtsH-mediated proteolysis in *E. coli*. More specifically, WaaA (also called KdtA) was shown to be rapidly turned over in WT cells but is stabilized by temperature-sensitive FtsH mutants (117). Further *in vitro* characterization showed that FtsH was capable of directly degrading WaaA (117). LpxD was recently identified as an interactor of LapB and subsequent *in vivo* experiments indicated that it is similarly proteolyzed by FtsH (91). Like LpxC, LpxD turnover was elevated in stationary phase in an FtsH-dependent manner, suggesting that both LpxC and LpxD may be co-regulated under those conditions (91). The physiological impacts of misregulated LpxD and WaaA, however, are unknown. As such, it is unclear whether FtsH-mediated proteolysis of these substrates serves to control production of lipid A or some other function.

Finally, there is some evidence that the activity of the membrane embedded enzyme LpxK might be regulated in *E. coli* under certain conditions. In particular, *in vitro* LpxK activity has been reported to elevate in the presence of phospholipids, particularly cardiolipin (118). However, it is unclear whether this stimulation constitutes a regulatory response or is simply a result of increased solubility of the hydrophobic LpxK substrate (118). Perhaps consistent with this, *E. coli* mutants deficient in cardiolipin exhibit reduced LPS production, but whether this is mediated through reduced LpxK activity is unknown (80). Additional evidence that LpxK might be regulated comes from the observation that lysates of a temperature-sensitive LpxD mutant have reduced LpxK specific activity (119). However, the mechanism by which LpxD activity might influence LpxK was unclear. More recently, computational modeling has suggested that LpxK activity might be stimulated by unsaturated fatty acids and that the impact of LpxD is indirectly due to an accumulation of LpxD substrate, *R*-3-hydroxyacyl-ACP, that feeds into saturated fatty acid biosynthesis (76). Nevertheless, the molecular mechanism by which unsaturated fatty acids might impact LpxK as well as the potential physiological impacts of LpxK misregulation are unknown.

## Outlook

Since the first report of regulated LPS biosynthesis in 1993, impressive strides have been made to understand how bacteria control flux through the lipid A biosynthesis pathway. The extensive characterization of LpxC regulation in *E. coli*, in particular, has led to a comprehensive model of regulated LPS synthesis that has revealed foundational principles that are broadly applicable to diverse bacteria. Nevertheless, many questions remain unanswered. It has been well established that the misregulation of LpxC has pleiotropic impacts on cellular physiology, but the reports of regulation of other enzymes in the lipid A biosynthetic pathway raise the possibility that additional layers of control may fine tune the production of LPS, which must be explored in future work. In addition, a deeper understanding of how lipid A biosynthesis is controlled in bacteria outside of the Enterobacteriaceae family is vital for the effective design and implementation of antibiotics that target the pathway. Further characterization of alternative model systems such as *P. aeruginosa* and *N. meningitidis*, in which LpxC does not appear to be inhibited by FtsH, have the potential to reveal new paradigms for the regulation of lipid A production. More specifically, uncovering the molecular basis for LpxC activation, the signal(s) that influence its regulation, and the physiological impacts of misregulation in these organisms will broaden our knowledge of the diverse strategies used to control the essential lipid A biosynthetic pathway, providing critical insight into therapeutic development.

Recent studies have underscored that the specific mechanisms used to regulate LpxC are not generalizable to all Gram-negative bacteria and, instead, phylogenetically distinct organisms have evolved unique mechanisms to control the production of LPS. Indeed, even in the close *E. coli* relative *S.* Typhimurium, slight deviations from the YejM-LPS-LapB partner switching model have been suggested (87). Due to the diversity of reported LpxC regulators and their lack of broad conservation, it is likely that as-yet-unknown strategies to control LPS biosynthesis exist within the Gram-negative bacterial clade. In particular, the critical priority pathogen *A. baumannii* does not appear to control LpxC activity through any of the known regulatory mechanisms, despite its close relatedness with *E. coli* and *P. aeruginosa*. Intriguingly, lipid A is not essential in *A. baumannii*, making it possible that the careful control of the lipid A biosynthesis pathway observed in other species is not necessary in this organism (44). Nevertheless, the negative effects associated with overproduction of lipid A are likely to still apply to *A. baumannii*. In addition, very little is known about how lipid A biosynthesis is regulated in species outside of the Proteobacterial phylum, increasing the likelihood that an array of regulatory strategies remains to be uncovered. Thus, future work in which the regulation of LPS biosynthesis is characterized in nontraditional model organisms with diverse lifestyles and physiologies will be critical to understanding the selective pressures and varied mechanisms associated with controlled production of lipid A.

## Conflict of interest

The author declares that they have no conflicts of interest with the contents of this article.
